# Planetary Health and Hospitals’ Contribution—A Scoping Review

**DOI:** 10.3390/ijerph192013536

**Published:** 2022-10-19

**Authors:** Lara Schmidt, Sabine Bohnet-Joschko

**Affiliations:** Chair of Management and Innovation in Health Care, Faculty of Management, Economics, and Society, Witten/Herdecke University, 58448 Witten, Germany

**Keywords:** climate change, public health, health impacts assessment, carbon footprint, greenhouse gas emissions, hospital, digital transformation

## Abstract

Climate change is one of the greatest global threats for planetary and human health. This leads to new challenges for public health. Hospitals emit large amounts of greenhouse gases (GHG) in their healthcare delivery through transportation, waste and other resources and are considered as key players in reducing healthcare’s environmental footprint. The aim of this scoping review is to provide the state of research on hospitals’ carbon footprint and to determine their contribution to mitigating emissions. We conducted a systematic literature search in three databases for studies related to measurement and actions to reduce GHG emissions in hospitals. We identified 21 studies, the oldest being published in 2012, and the most recent study in 2021. Eight studies focused on GHG emissions hospital-wide, while thirteen studies addressed hospital-based departments. Climate actions in the areas of waste and transportation lead to significant reductions in GHG emissions. Digital transformation is a key factor in implementing climate actions and promoting equity in healthcare. The increasing number of studies published over time indicates the importance of the topic. The results suggest a need for standardization of measurement and performance indicators on climate actions to mitigate GHG emissions.

## 1. Introduction

Climate change is one of the greatest global challenges to public health [1] and is indeed an existential threat for human health in two ways. First, by changing the severity or frequency of health problems that are already affected by climate or weather factors, and second, by creating unprecedented or unanticipated health threats in places where they have not previously occurred [2]. The impacts of climate change affecting both planetary and human health are projected to increase in intensify throughout this century. The carbon footprint is an important factor to express these impacts and to measure the amount of greenhouse gases released into the atmosphere by a given activity [3,4]. Climate-related disasters lead to direct and indirect health impacts regarding morbidity and mortality. The health impacts of climate change can be described in three groups, which are 1. Direct impacts of climate and weather, 2. Ecosystem-mediated impacts of climate change and 3. Health impacts through human institutions.

1. Direct impacts of climate and weather refer to direct illnesses and deaths due to more frequent extreme climate-related weather events. These include rising temperatures and droughts leading to heat stress and cardiovascular failure [5] and an increased frequency and intensity of storms and floods causing mortality and morbidity [3,6,7].
2. Ecosystem-mediated impacts of climate change include disease and mortality caused by indirect effects of climate change, due to variations in the ecosystem [5]. Changes in temperature and intensified precipitation and humidity lead to deterioration in water quality and quantity, resulting in an increased risk of waterborne and infectious diseases [8,9,10,11]. Shifting ecosystems lead to a deterioration in food and nutrition security [12]. Deterioration in air quality, particularly due to temperature increases and wildfires, results in increased respiratory diseases, cardiovascular disease and cancer [5].
3. Health impacts through human institutions include diseases and mortality from altered systems created by humans [5]. The level of physical exertion by agricultural and construction workers caused by rising temperatures, particularly in tropical developing countries, leads to higher health risks. In contrast, the development of an urban environment with a simultaneous reduction in green spaces results in increased respiratory diseases caused by increased air pollution [13].

Crises such as the COVID-19 pandemic reinforce the need for ecosystem protection and a better understanding of environmental-health dynamics [5]. Opportunities to both mitigate climate change by reducing greenhouse gas emissions and protect health have received increasing attention in recent years [14]. The Paris Agreement, as the first universal and legally binding global climate agreement, enacted in 2016, established an initial framework to reduce the impacts of climate change and support countries in their effort to mitigate emissions and to manage the climate crisis [15]. The Intergovernmental Panel on Climate Change ‘1.5 Degree Report’ from 2018 highlights the potential consequences of climate change if no actions are taken to mitigate CO_2_ emissions [16]. For healthcare delivery, the concept of Planetary Health is becoming increasingly important worldwide [17,18]. The term “Planetary Health” emerged from the environmental and holistic health movements of the 1970–1980s. Following the Rockefeller Foundation–Lancet Commission on Planetary Health report, the concept has penetrated the academic and medical discourse [19]. The concept defines the achievement of the highest attainable standard of health, well-being and equity. It can be reached through a global consideration of the human systems that shape human futures, such as politics, economics and society, as well as the Earth’s natural systems [20].

Healthcare delivery itself has also been shown to contribute to climate change [21]. The modern healthcare sector is among the ‘heavy-emitting’ sectors, accounting for about 4–5% of global GHG emissions [3,22]. In particular, hospitals are highly energy intensive, produce substantial waste and consume large amounts of resources in their service delivery [23]. By taking actions to reduce GHG emissions, hospitals are key players in mitigating climate change. Fundamental climate actions such as strategies to reduce waste, avoid transportation and purchase sustainable products and chemicals can be mentioned as steps towards improving the climate [24,25]. The Global Green and Healthy Hospitals (GGHH) network, recognized by the World Health Organization (WHO), targets to promote greater sustainability and environmental health in the health sector. To achieve this, a framework was created consisting of 10 interconnected Sustainability Goals. These cover the following areas:

- **Leadership:** Leadership support in order to achieve a long-term organizational culture in which environmental health becomes a strategic priority.
- **Chemicals:** Protecting and improving the health of patients, employees and the environment by using safe chemicals, materials, products and processes that exceed the requirements of environmental regulations.
- **Waste:** Reducing, treating and disposing of medical and non-medical waste in an environmentally sound manner.
- **Energy:** Promoting energy efficiency while reducing fossil fuel use.
- **Water:** Reducing water consumption and wastewater pollution in the hospital and providing potable water.
- **Transportation:** Improving transportation strategies for patients and staff to reduce local pollution.
- **Food:** Promotion of healthy, locally and sustainably produced foods for patients and staff.
- **Pharmaceuticals:** Prescribing, safe management and proper dispose of pharmaceuticals.
- **Buildings:** Green and healthy hospital design and construction, e.g., by the use of high reflectance roofing and paving, or greening of roof but also promoting inhabitant choice and control regarding lighting and indoor air quality.
- **Purchasing:** Purchasing safer and more sustainable products and materials [26,27].

Building on this framework, our scoping review aims to map the current state of research on hospitals’ carbon footprint. Second, we want to analyze this research for reported climate actions based on the 10 GGHH Sustainability Goals.

## 2. Methods

The five-step methodological framework for scoping reviews defined by Arksey and O’Malley was adopted to map existing evidence on breadth and depth of research [28]. The review followed five steps: (1) identifying a research question, (2) identifying relevant studies, (3) study selection, (4) charting the data and (5) collating, summarizing and reporting results. We conducted the review process as follows.

### 2.1. Identification of the Research Question

Our aim was to create a general survey of existing studies by conducting a scoping review on hospitals ‘contribution to the carbon footprint and identifying research gaps. We stated our research question as “How do hospitals contribute to reducing the carbon footprint?”

### 2.2. Identification of Relevant Studies

According to Arksey and O’Malley, comprehensiveness and breadth is important in the search [28]. We conducted database searches in PubMed, Science Direct and EBSCO from January 2022 to March 2022. These databases were selected for the review because they contain relevant studies about healthcare’s carbon footprint. PubMed covered the main part of the literature. The search string consisted of terms considered by the authors to describe hospitals´ contribution to reducing the carbon footprint. We have focused on hospitals as large and relevant healthcare organizations, which are considered to be important actors in the implementation of actions to reduce the carbon footprint [23]. The search process occurred iteratively. The used search terms covered all areas of the carbon footprint and hospitals, including Medical Subject Headings (MeSH) terms, subject headings and keywords. The search string was tailored to the specific requirements of each database. The corresponding search syntaxes for each database are outlined in Table 1.

### 2.3. Selection of Studies

**Figure 1:**
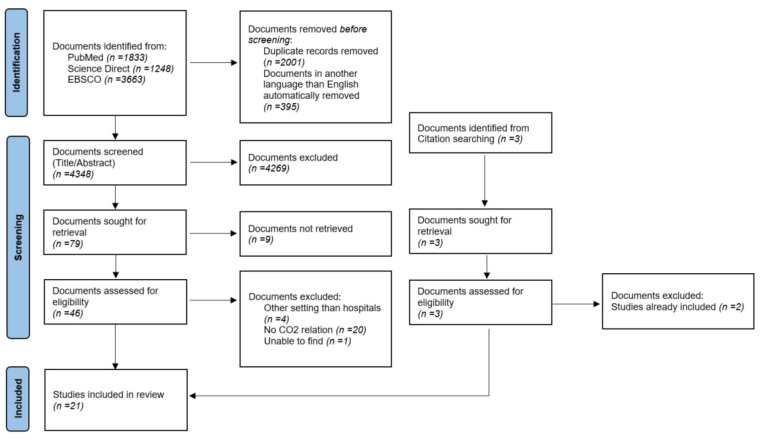
Preferred Reporting Items for Systematic Reviews and Meta-Analyses (PRISMA) flow diagram for articles identified, screened and included in the review.

To categorize and answer our research question, we followed the standard procedure of PRISMA [29]. Criteria for inclusion and exclusion were defined a priori and are outlined in Table 2. The study selection was performed by two independent reviewers, based on title, abstract and full text examination. Differing assessments regarding the interpretation of inclusion and exclusion criteria were discussed until a consensus was reached. For full-text eligibility, the same criteria were applied. The study selection is presented using a PRISMA flow diagram in Figure 1 [29,30].

### 2.4. Charting the Data

After identifying the articles that are relevant for the review, the primary author extracted the data into an evidence table. Data were summarized by lead author, journal and year of publication, article type and research objective, country of origin, type of methodology used and the hospital-based department, where each study was conducted. In addition, the t10 Sustainability Goals are outlined. Other characteristics include the type of measurement as well as a description of climate actions implementation and impacts.

### 2.5. Reporting the Results

To ensure the accuracy of the information and complete coverage of the article results, an independent reviewer screened the evidence table. We analyzed the main data from the included studies, by group or topic when possible. A quality assessment of individual articles was not necessary [28] as every publication came from a peer-reviewed journal. A summary of the results obtained is provided below.

## 3. Results

Our database search identified 4743 articles, after removing duplicates and studies not written in English. Of these, 4269 articles were excluded based on title or abstract. We reviewed 79 studies for full-text eligibility, of which 20 met our inclusion criteria. A manual screening of the reference lists of the included studies identified one more study. In total, we identified 21 studies for this scoping review.

### 3.1. Study Characteristics

**Figure 2:**
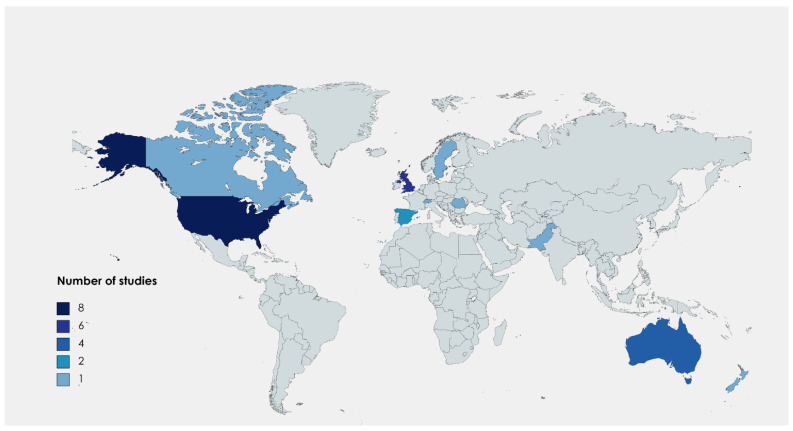
Map of included studies.

The 21 studies that met our eligibility criteria were released in 19 different peer-reviewed journals with an impact factor. The journals can be attributed to two main research fields: (1) environmental and natural science and (2) medical and health science. The oldest study was published in 2012, and the most recent was published in November 2021. The majority (*n* = 8) were published in 2021. The included studies originated from the United States (*n* = 8), United Kingdom (UK) (*n* = 6), Australia (*n* = 4), Spain (*n* = 2) Pakistan and Sweden, Switzerland, Canada, New Zealand and Romania with one study each (Figure 2). Three studies conducted international comparisons of hospital-based departments. One study focused on the nations of Canada, the United States and the United Kingdom [31]. Another study focused on the nations Australia, the United States and the United Kingdom [32]. A third study compared two hospital-based departments in Australia and the United States [33].

The studies included both a quantitative and a qualitative methodical approach. None of the studies utilizing a purely qualitative approach, while 18 studies followed a full quantitative approach [31,32,33,34,35,36,37,38,39,40,41,42,43,44,45,46,47,48]. Three studies applied a mix of quantitative and qualitative methods [49,50,51]. All studies reported their footprint in GHG emissions, of which the CO_2−_ footprint is a major component.

### 3.2. Hospitals’ Focus and Sustainability Goals

Thirteen of the referenced studies highlighted different hospital-based departments that emitted high amounts of greenhouse gas. Of particular note are operating theatres, investigated in four studies [34,47,48,50]. Two studies analyzed GHG emissions in pathology [39,40], while two other studies focused on hospital kitchens and the associated meal menus for patients [42,43]. Other hospital-based departments included radiology [35], orthopedics [45], rehabilitation [31], intensive care unit [33] and neurology [32], with one study each. Eight studies analyzed GHG emissions hospital-wide [36,37,38,41,42,46,49,51]. National comparisons were conducted in seven of the referenced studies. Of these, five studies analyzed a hospital as a whole [36,38,46,49,50], and two focused on a hospital-based department [34,40]. International comparisons were performed in the hospital-based department intensive care units [33] and operating theatres [47,48]. We combined the hospital’s focus with the 10 Sustainability Goals of the GGHH agenda, shown in the following matrix (Table 3).

In terms of the Sustainability Goals, it is noticeable that 9 of 10 Sustainability Goals are represented in the existing research literature. Most studies focused on the Sustainability Goals: energy (*n* = 17), transportation (*n* = 15) and waste (*n* = 14). The areas of purchasing (*n* = 6), water (*n* = 6), chemicals (*n* = 4), pharmaceuticals (*n* = 4) food (*n* = 3) and buildings (*n* = 2) can also be identified. The studies covered more than one Sustainability Goal each. The leadership Sustainability Goal is not presented in the existing literature.

### 3.3. Study Design

We categorized the included studies into three study types. Eleven of the studies were observational studies [33,34,35,38,40,41,42,43,47,49,50]: Eight of these studies focused on the measurement of GHG emissions in different hospital-based departments [33,34,35,40,42,43,47,50]. Three studies measured the GHG emissions hospital-wide [38,41,49]. Five observational studies compared GHG emissions nationally [34,38,40,41,49] two studies compared GHG emissions internationally [33,47].

Two additional studies simulated scenarios for implementing climate actions to reduce GHG emissions [48,51]: One study focused on actions to reduce GHG emissions from wastes by combining disposable and reusable products [48], while the other simulated different scenarios for waste incineration [51].

The third type of study centered the effective implementation of climate actions. In total, eight studies implemented climate actions. These used, inter alia, before/after models to illustrate the results of implementing their actions [31,32,36,37,39,44,45,46].

### 3.4. Measurement of Greenhouse Gas Emissions

Eleven studies focused on measuring GHG emissions from hospitals and their departments. The majority of numerical data were reported as total emissions and CO_2_ equivalents. All studies collected primary data. These included, for example, sorting and weighing waste [42,50], and measuring the energy consumption [34,50]. A survey of patients and staff was used to determine the distance of travel [50]. Secondary data were collected from hospitals [33,49] and manufacturers of relevant products [49]. One study classified building energy classes [41].

Life cycle assessment (LCA) has an essential part regarding the measurement of GHG emissions of a product. Eleven studies used a LCA. Four of these focused on the measurement of GHG emissions [33,35,38,43], seven additional studies implemented climate actions [31,36,37,40,46,48,51].

National and international databases were used to calculate GHG emissions from the collected data [34,35,40,42,47,49,50]. One study used a computer-based software to analyse GHG emissions [35].

### 3.5. Description of Hospitals’ Climate Actions

The implementation of climate actions is an important step in reducing GHG emissions. Four studies focused on the transportation Sustainability Goal [31,32,44,45]. Climate actions included replacing physical appointments of patients with telemedical consultations. These actions addressed the hospital-based departments orthopedics [45], rehabilitation [31] and neurology [32]. Another study considered a single hospital [44]. One study conducted a telephone patient survey. Patients were asked about their preferences for future consultations as well as the actual type of transportation. The subsequent data collection of GHG emissions was based on the greenhouse gas report from 2020 [45]. Three other studies calculated the travel distance to the hospital and the associated GHG emissions based on the patients’ residential data [32,44,50]. Two of these also considered the additional GHG emissions generated by the use of telemedicine services for the respective hospital-based department [31,32]. All studies reported reductions in GHG emissions through the implementation of climate actions. Despite the increased use of digital technologies, these actions also estimated a reduction in carbon costs in the areas of energy and waste [32,45].

In total, four studies considered climate actions in the area of waste [36,37,39,46]. Three of these dealt with actions to reduce waste by switching from disposable containers (DSC) to reusable sharps containers (RSC). The implementation of these climate actions addressed 5 hospitals [46], 40 interrelated hospitals [36], as well as the implementation in a single hospital [37]. In the latter study, already existing RSC were also adapted in addition to converting RSC [37]. Primary and secondary data were collected to calculate GHG emissions. These included data from national databases [36,37,46], industry data [36] and primary activity data [36,37]. The implementation of climate actions resulted in a reduction in GHG emissions [36,37,46].

A fourth study focused on the hospital-based pathology department. Non-urgent pathology testing was limited to two days per week to reduce waste. Primary data, such as patient referral data, as well as secondary data from previous studies were used to calculate GHG emissions. The implementation of climate actions led to a reduction in GHG emissions [39].

Two studies conducted scenarios that represented different cases of possible implementation of climate actions. Both implemented actions in waste management [30,51]. One scenario focused on a single hospital [50] and the other on the hospital-based operating theatre department [48]. One study used a spreadsheet. These concluded that an integrated waste management through various techniques of composting, incineration and recycling had the lowest GHG emissions compared to the incineration and landfilling of waste. Transport routes and energy emissions from waste incineration were also considered [51]. The second study modelled different combinations of disposable and reusable product use for surgery in hospitals in three different countries. The hospitals in the United States and the United Kingdom reduced GHG emissions when switching from single-use to reusable products. The Australian hospital increased its carbon footprint [48].

## 4. Discussion

As the climate becomes more unstable and less predictable due to environmental changes, there are significant implications for planetary and human health. According to the WHO, the health sector has an essential part in reducing the carbon footprint, while strengthening the health systems to respond to the impacts of climate change [24]. A number of co-benefits of climate change mitigation for health can be achieved through, for example, reduction in the environmental burden of disease, reduction in years of life lost and fewer hospitalizations [52]. This scoping review provided new information about the hospitals’ contribution reducing their carbon footprint in healthcare delivery.

Research in the area of carbon footprint has increased over the past 10 years. This period coincides with increasing public interest in the impact of climate change on health, the publication of sustainable development goals and frameworks and increasing public policy discourse on planetary health. The increased focus is also explained by global agreements such as the Paris Agreement [15] or the European Green Deal, enacted in 2021 [53].

Considering the geographic scope analyzed in Section 3.1, a possible direction for future research becomes apparent. The included studies were conducted in 10 countries. A high number of studies from the United States and the United Kingdom is evident. The healthcare system of the United Kingdom, the National Health Service (NHS), started setting national targets to reduce carbon emissions, according to the Greenhouse Gas Protocol, in 2008. The target is an 80% reduction for the NHS footprint by 2040 (compared with a 1990 baseline) [54]. In the United States, the maintenance organization Kaiser Permanente, as one of the largest nonprofit healthcare plans, has achieved its goal of becoming carbon-neutral in 2020 by implementing a number of climate actions [55]. This indicates that the implementation of climate actions is feasible in both government and privately managed systems. Due to the different healthcare systems and a lack of key indicator systems, generalizability to other healthcare systems is limited.

Regarding the Sustainability Goals of the GGHH network (Section 3.2), we were able to identify relevant aspects of hospitals’ carbon footprint. The studies particularly address energy, waste and transportation, these Sustainability Goals being considered in about 90% of the existing literature. Most studies deal with more than one sustainability goal, substantiating the complexity and interrelation of climate actions and goals. For example, one study gives evidence that reusable products will reduce waste but not necessarily GHG emission due to an increase in water, energy and transport emissions for washing and additional transportation [48]. Moreover, the GGHH framework allows to identify a substantial research gap in the existing literature on hospitals’ carbon footprint. In total, our scoping review identified research on nine of ten Sustainability Goals but none on ‘leadership’. However, leadership is essential in order to foster green and healthy hospitals [27] and will be the first instance for planning and implementing climate actions and creating an ecologically sustainable corporate culture [56]. Key organizational priorities therefore include environmental health, safety and sustainability [27]. Hospital leadership often means physician leadership and includes top-down strategic approaches as well as individual and departmental initiatives.

Concerning the implementation of climate actions in the hospital-based departments, operating theatres [34,47,48,50] and pathology [39,40], in particular, can have a significant impact on reducing GHG emissions in hospitals. These hospital-based departments emit a high amount of greenhouse gases.

Regarding techniques for measurement (Section 3.4), most studies deal with the measurement of carbon footprint in different hospitals and their departments [33,34,35,38,40,41,42,43,47,49,50]. An important step in measuring GHG emissions is the LCA. By gathering data from life cycle assessment databases, the studies are able to quantify the product from manufacturing to disposal and to analyze the life cycle. In this context, the life cycle inventory has been used [33,35,38,43]. Further literature underlines the relevance of life cycle assessment for the calculation of GHG emissions [8].

Other studies are taking a step further by implementing initial climate actions to reduce their carbon footprint. A detailed description of these can be found in Section 3.5. These climate actions are particularly related to the Sustainability Goals of transportation [31,32,44,45] and waste [36,37,39,46].

Climate actions to reduce waste focus on switching from single-use containers to reusable sharps containers [36,37,46]. According to current literature, converting to reusable equipment is likely to have positive economic and ecological effects [48]. Thus, all studies conclude that healthcare facilities can reduce waste and emissions through composting, recycling, better procurement and minimizing waste transportation [36,37,46]. Other articles reinforce the relevance of implementing actions in waste management. These highlight that 15% of waste generated in a healthcare facility is dangerous waste [57], which can be harmful to both humans and the environment [58].

The implementation of climate actions in transportation refers to the replacement of physical visits with telemedical consultations. Due to the increasing digital transformation in the healthcare sector, this action can be implemented with greater ease. In addition to reducing GHG emissions, this can also lead to reduced time and costs for patients and staff. [38]. Besides the benefit of environmental sustainability, telemedicine can offer more efficient and equitable access to healthcare [52].

Our scoping review shows evidence for the positive impact of climate actions. Standardized key performance indicators and the use of uniform databases are highly relevant for the documentation of greenhouse gases and are a prerequisite for the comparison and generalizability of climate actions.

This scoping review has some limitations. Although our search included three common databases (i.e., PubMed, Science Direct and EBSCO), we may not have identified all studies in the published literature. The search strategy and the applied inclusion and exclusion criteria may have missed articles using different definitions or terminology. We only included studies in English. The decision to exclude non-English studies followed the expectation that research on this global topic would be published primarily in English. The included studies focus on developed nations, so our results are not representative for all regions and countries. In addition to the limitations, the strengths of the study can be mentioned. We used a transparent and reproducible process conducted by two independent researchers. The framework used for analysis covers all relevant Sustainability Goals. This provides a general survey of the young but increasingly important topic. Our review also identified research gaps that can provide guidance for future research.

## 5. Conclusions

This scoping review provides a general survey of hospitals’ contribution to the carbon footprint. Most research has been devoted to assessing the measurement of GHG emissions in hospitals and their departments, while fewer studies have included the effective implementation of climate actions to reduce the carbon footprint. The implementation of digital technologies in the form of teleconsultations contributes to climate protection and to an improved and more equitable access to healthcare. It illustrates that high-quality patient care and an environmentally sustainable healthcare organization do not have to be mutually exclusive. The majority of the studies originate from large countries where providers have already implemented climate actions to reduce their carbon footprint. The results cannot be generalized due to lack of standardized key performance indicator systems. Developing these is an essential challenge for research and practice and could be fostered and stimulated by politics as well. Further political implications include awareness management regarding climate protection in the hospital sector and could provide guidelines for hospitals’ use of energy and waste. Future research should aim to analyze the contribution of physician leadership and hospital management to mitigate GHG emission. Hospital leadership will gain importance to achieve global and long-term reductions in hospitals’ GHG emissions and contribute to planetary health.

## Figures and Tables

**Table 1:** Search string.

Databases	Search String
PubMed	(“Hospitals”[Mesh]) AND “Carbon Footprint”[Mesh] OR (hospital[Title/Abstract]) AND (carbon neutral[Title] OR carbon footprint[Title] OR emission*[Title] OR green*[Title] OR sustainab*[Title]
Science Direct	hospitals AND (“Carbon footprint” OR “Carbon neutral” OR “emission” OR “greenhouse” OR sustainab”)
EBSCO	AB ( (MM “Carbon Footprint”) AND (MM “Hospitals+”) ) OR AB hospital AND TI ( carbon footprint OR carbon neutral OR emission* OR green* OR sustainab*)

**Table 2:** Inclusion and exclusion criteria for scoping review.

Inclusion Criteria	Exclusion Criteria
■ Studies published in English ■ Empirical Studies ■ Peer-reviewed articles ■ Articles that provide insights into CO_2_ emissions in hospitals ■ Studies illustrating CO_2_ measurements in hospitals ■ Studies illustrating CO_2_ mitigation actions in hospitals	■ Studies published in languages other than English ■ Studies that are not empirical ■ Studies that are not peer reviewed ■ Studies focused on environmental diseases, sustainability of medical treatments, sustainability in non-ecological context ■ Studies conducted in settings other than hospitals

**Table 3:** Matrix of Sustainability Goals (GGHH) and hospitals’ focus.

Goals		Leadership	Chemicals	Waste	Energy	Water	Transportation	Food	Pharmaceuticals	Buildings	Purchasing
	Focus										
Hospital-based departments	Operating theatre		[47,48]	[34,47,48,50]	[34,47,48,50]	[48]	[34,47,50]		[34,48]		[34,48,50]
	Pathology			[39,40]	[40]	[40]	[40]				
	Radiology		[35]	[35]	[35]	[35]					
	Orthope-dics				[45]		[45]				
	Rehabili-tation				[31]		[31]				
	Intensive care unit			[33]	[33]						
	Neuro-logy				[32]		[32]				
	Hospital kitchen					[42]		[42,43]			
Single hospital	Single hospital			[36,37,38,46,49,50]	[36,37,38,41,46,49,51]	[36,38,42]	[36,37,46,49,51]	[42]	[38]	[38,41]	[36,37,46]
Hospitals and departments in comparison	National		[40]	[34,36,38,40,46,49,50]	[34,36,38,40,46,49,50]	[34,36,38,40,46,49]	[34,36,40,46,49,50]	[38]	[34,38]	[38]	[34,36,40,46,50]
	Interna-tional		[47,48]	[33,47,48]	[33,47,48]	[48]	[47]		[33,47]		[48]

## Data Availability

Not applicable.
